# Varus-valgus native knee laxity in extension displays an almost rectangular pattern and does not correlate with lower limb alignment

**DOI:** 10.1371/journal.pone.0313402

**Published:** 2024-11-12

**Authors:** Manabu Akagawa, Hidetomo Saito, Yasuhiro Takahashi, Hiroaki Tsukamoto, Kazunobu Abe, Yosuke Iwamoto, Takayuki Yoshikawa, Toshiki Abe, Hiroaki Kijima, Yuji Kasukawa, Koji Nozaka, Naohisa Miyakoshi

**Affiliations:** 1 Department of Orthopedic Surgery, Akita University Graduate School of Medicine, Hondo, Akita, Japan; 2 Department of Orthopedic Surgery, Omagari Kousei Medical Center, Daisen, Akita, Japan; 3 Department of Orthopedic Surgery, Noshiro Kousei Medical Center, Noshiro, Akita, Japan; San Raffaele University Milan, ITALY

## Abstract

Optimal ligament balance is crucial for successful total knee arthroplasty (TKA), which is a standard procedure for managing end-stage knee osteoarthritis. However, the reported values of ligament balance vary due to different methodologies and population differences, which requires further studies. This study aimed to investigate the varus-valgus ligament balance in healthy knees of Japanese individuals and its relationship with lower limb alignment. The studyanalyzed64 knees from 33 healthy young adults using stress radiographs to measure varus-valgus laxity. The mechanical hip-knee-ankle angle, medial proximal tibial angle, and lateral distal femoral angles were determined using digital long-leg radiographs. Varus and valgus laxities were compared, and the relationship between ligament balance and alignment parameters was analyzed. Mean varus laxity (3.6°± 1.3°) was significantly greater than mean valgus laxity(2.9°± 1.0°) (*p*<0.0001). No significant association was observed between ligament balance and alignment parameters. Healthy knees of Japanese individuals exhibited slightly greater varus laxity (0.7°) than valgus laxity, with no correlation observed between ligament balance and lower limb alignment. The finding underscores the importance of recognizing alignment diversity while noting that alignment does not influence ligament balance; this is particularly relevant in modern TKA techniques focusing on patient-specific joint and ligament reconstructions. The study’s findings could help formulate strategies benefiting knee osteoarthritis management.

## Introduction

Total knee arthroplasty (TKA) is a common procedure for managing end-stage knee osteoarthritis (OA). Ligament balance in TKA is recognized as a crucial factor influencing postoperative outcomes [1, 2]. Traditional mechanical alignment TKA aims to create a rectangular ligament balance in extension and flexion [3]. However, achieving equal rectangular gaps for extension and flexion is challenging. Recent study have suggested a certain tolerance for laxity on the lateral side [4]; however, the acceptable extent of this laxity is unclear, and the boundary between laxity and instability remains undefined. Kinematic alignment TKA has recently focused on reconstructing the pre-arthritic joint without releasing the ligaments [5]. The caliper technique allows the reconstruction of patient-specific alignment and ligament balance [6]. Thus, the goals of ligament balance in TKA have evolved, particularly toward achieving a more physiological ligament balance.

Several studies have investigated ligament balance in healthy knees to understand physiological ligament balance. A recent meta-analysis of *in vitro* studies revealed that the joint asymmetry of varus-valgus laxity during extension was significant; however, the degree was only 0.17° [7]. In contrast, a Japanese *in vivo* study revealed a varus laxity 2.5° greater than valgus laxity [8]. Direct comparison of results from previous studies is difficult because of the heterogeneity in methodologies, including variations in stress radiographs, applied loads, measurement units, participant demographics (such as age), racial diversity, and the exact nature of the physiological ligament balance.

Regarding racial differences, a previous study revealed greater anterior cruciate ligament laxity in Japanese individuals than in Caucasians, along with distinctive bone morphology characteristics, such as femoral valgus and tibial varus [9]. Furthermore, Japanese lower limb alignment tends to be more varus than that in Western populations [10]. However, the impact of such distinctive bone morphology and racial differences on varus-valgus laxity remains unclear.

This study primarily aimed to investigate the varus-valgus ligament balance in the extension of healthy knees of Japanese individuals. The secondary aim of the study was to explore the relationship between lower limb alignment and ligament balance. We hypothesized that, in healthy knees of Japanese individuals, the varus laxity in extension would be significantly greater than valgus laxity and that an association exists between lower limb alignment and ligament balance.

## Materials and methods

### Participants

**Fig 1:**
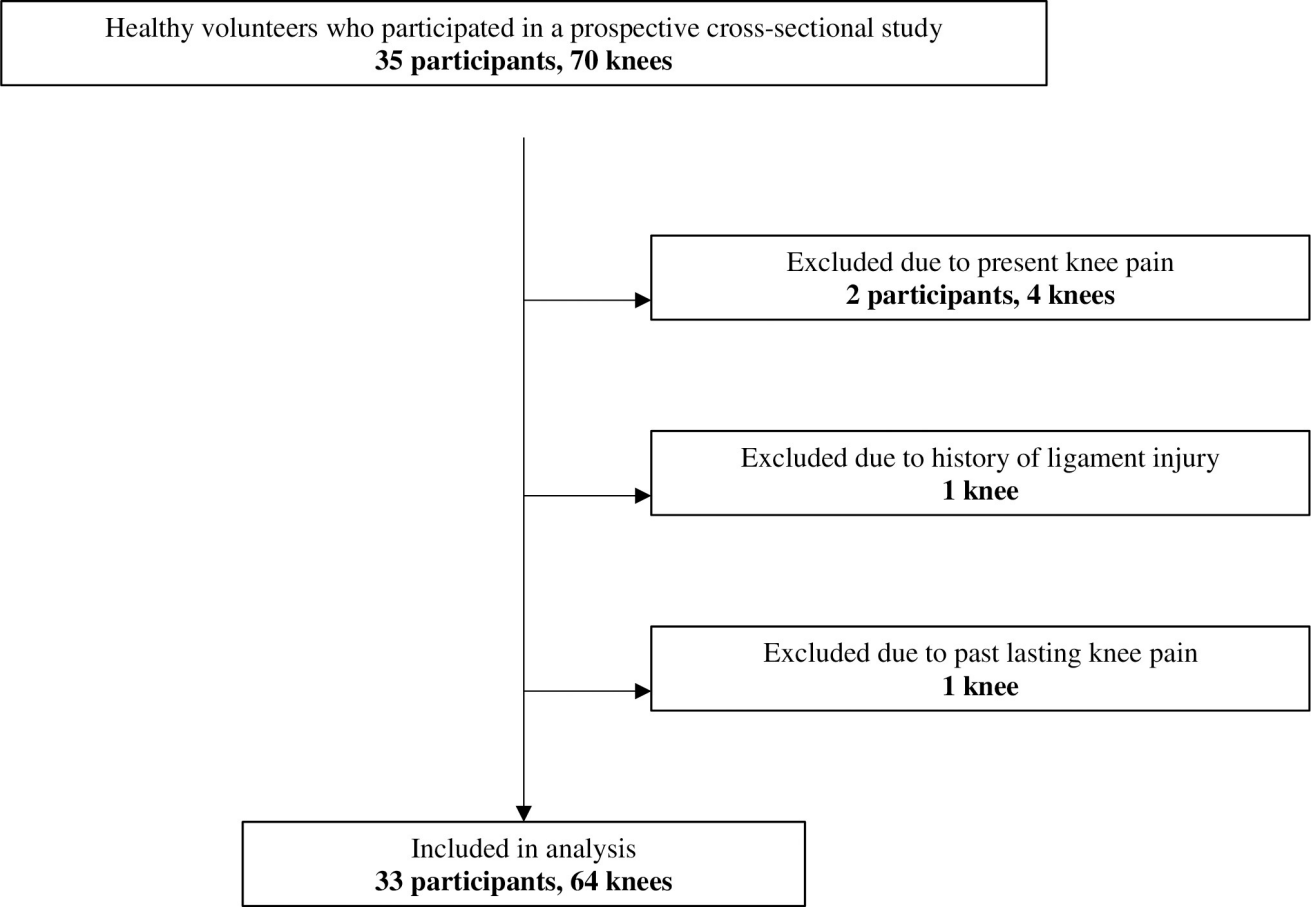
Flow chart of inclusion and exclusion criteria. The flowchart illustrates the sequential steps of inclusion and exclusion of participants in the study.

The healthy young adult cohort comprised in-hospital volunteers who participated in a prospective cross-sectional study conducted between March 10th and April 20th, 2023. This study enrolled 35 participants (70 knees). The following exclusion criteria were used to define healthy knees: participant age over 60 years; obvious osteoarthritic changes (Kellgren–Lawrence grade > 1); history of knee surgery or trauma, including ligament injury and fracture; history of knee pain lasting for more than 1week; present knee pain; rheumatoid arthritis; excessive knee joint laxity, especially hyperextension; contracture or restricted range of motion. After applying these criteria, 33 participants (19 men and 14 women) with 64 knees were included in the study (Fig 1). Written informed consent was obtained from all participants, and the ethics committee of Omagari Kousei Medical Center approved this study (approved #22–044).

### Radiological analysis

Varus-valgus laxity in extension was assessed using a Telos stress device in the anteroposterior view of the knee. The Telos device was applied at 15daN at the joint-line level [11], with the knee angle at 0° flexion. The X-rays were directed anteriorly at a 10° angle from above to ensure the tibial plateau was perpendicular to the film. Knee angle and rotation were controlled by a radiologic technologist to prevent malrotation, and only radiographs where the tibia overlapped the fibula by one-third to two-thirds were accepted. The angle between the tangent line on the femoral condyles and the tibial plateau was measured on varus, valgus, and neutral radiographs. In this calculation, the lateral opening angle was defined as positive, and the medial opening angle was defined as negative. Relative varus laxity is the absolute value of the angular difference between the varus and neutral radiographs, and relative valgus laxity is the absolute value of the angular difference between the valgus and neutral radiographs.

Digital long-leg radiographs were obtained as described by Paley et al. [12]. The mechanical hip-knee-ankle angle (mHKA) was defined as the angle between the mechanical axes of the femur and tibia. The mechanical medial proximal tibial angle (MPTA) was the medial angle between the mechanical axis of the tibia and the joint line of the proximal tibia. The mechanical lateral distal femoral angle (LDFA) was defined as the lateral angle between the mechanical axis and the joint line of the distal femur.

### Statistical analysis

Before the study, G*Power analysis (Ver.3.1.9.6) showed that 64 (α = 0.05, 1-β = 0.8, effect size = 0.5) and 55 knees (α = 0.05, 1-β = 0.8, effect size = 0.15) were necessary to achieve statistical power for the primary and secondary aims, respectively. For the secondary aim, the null hypothesis was that there would be no significant association between lower limb alignment and ligament balance, whereas the alternative hypothesis was that a significant association does exist.

For the primary aim, the differences in relative varus and valgus were analyzed using paired t-tests. For the secondary aim, a single regression analysis was performed to investigate the relationship between the differences in relative varus-valgus laxity and age, sex, and alignment parameters. All statistical analyses were performed using EZR [13], and statistical significance was set at *p*<0.05.

## Results

**Fig 2:**
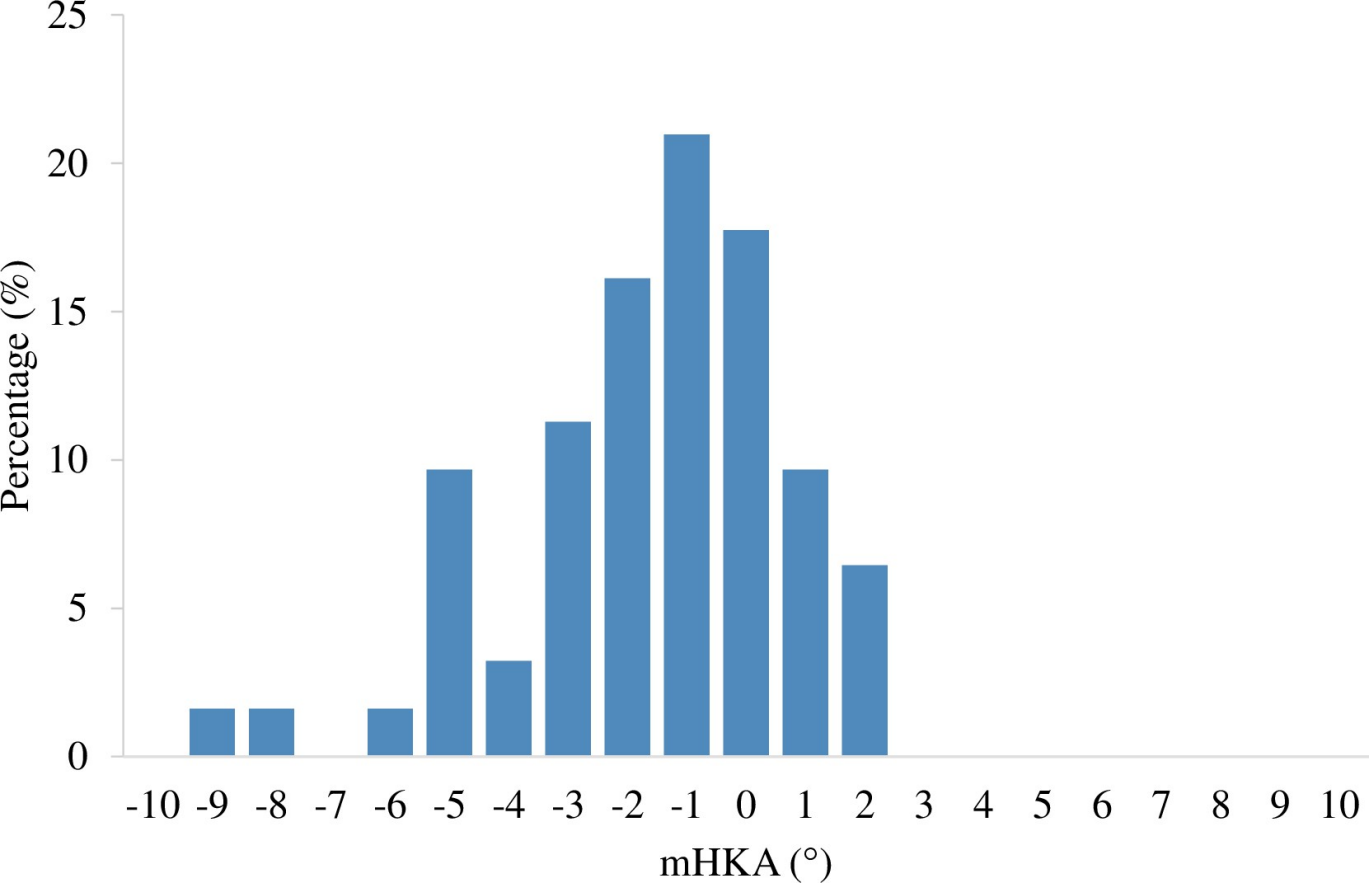
Distribution of lower limb alignment. The distribution of lower limb alignment in the native knees of Japanese individuals showed substantial diversity, with a maximum varus angle exceeding -9°.

Table 1 summarizes the participant characteristics and radiological parameters. The mHKA, MPTA, and LDFA values were -1.9°± 2.5°, 84.9°± 1.9°, and 86.1°± 1.9°, respectively. The distribution of mHKAwas diverse (Fig 2).

**Table 1:** Participants’ characteristics and radiological parameters.

**Sex (M: F)**	19:14
**Age (years)**	33.7 ± 9.8
**mHKA (°)**	-1.9 ± 2.5
**MPTA (°)**	84.9 ± 1.9
**LDFA (°)**	86.1 ± 1.9
**Absolute varus(°)**	4.2 ± 1.6
**Absolute valgus (°)**	-2.3 ± 1.0
**Neutral (°)**	0.6 ± 0.9
**Relative varus(°)**	3.6 ± 1.3
**Relative valgus (°)**	2.9 ± 1.0

mHKA: mechanical hip–knee–ankle angle; MPTA: medial proximal tibial angle; LDFA: lateral distal femoral angle; M, male; F, female

**Fig 3:**
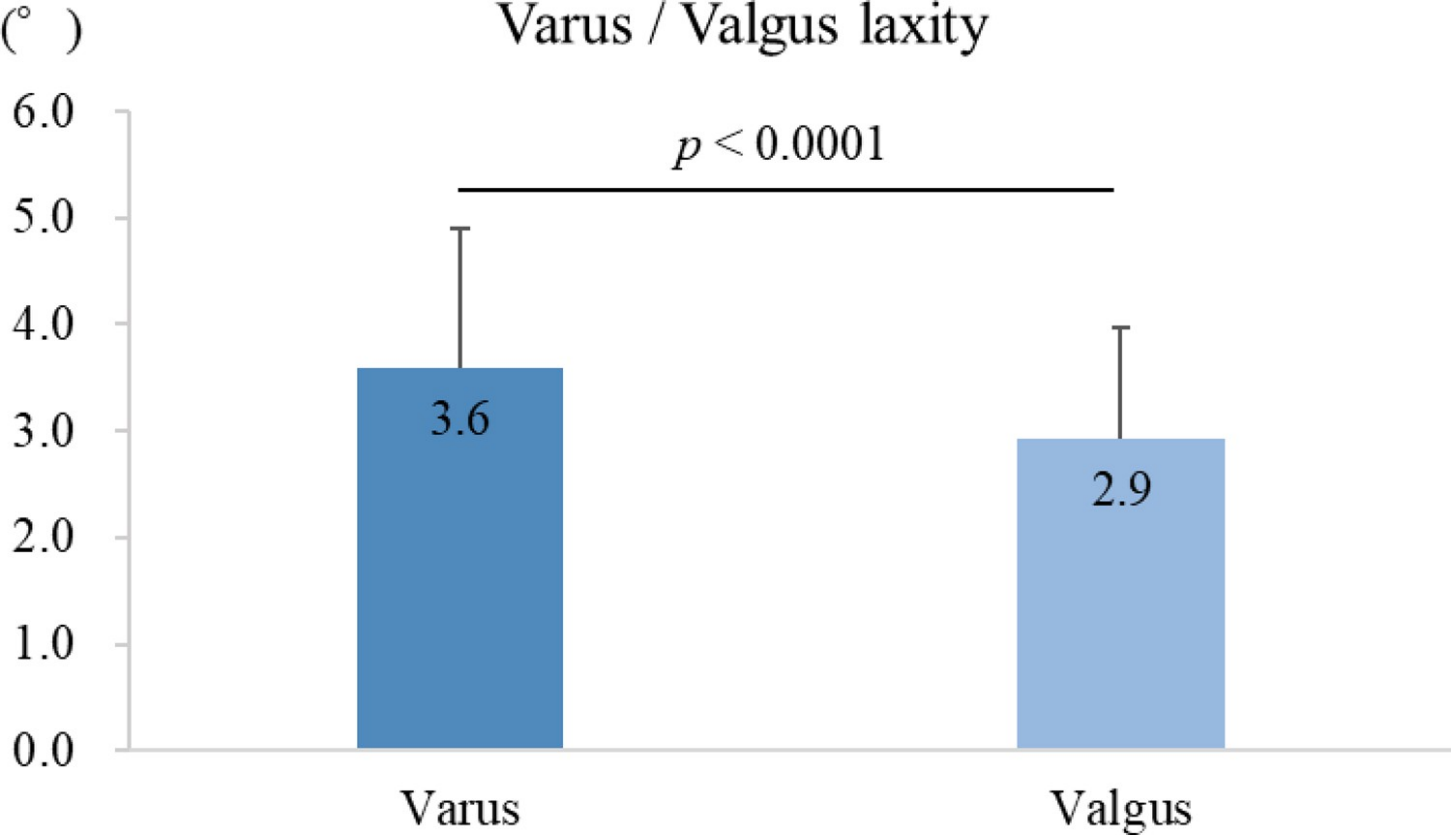
Varus and valgus laxity of the native knee. Varus laxity was significantly greater than valgus laxity by 0.7°.

The mean varus and valgus laxities were3.6°± 1.3° and 2.9°± 1.0°, respectively. Varus laxity was significantly greater than valgus laxity (*p*<0.0001) (Fig 3).

No significant association was observed between varus and valgus differences and age, sex, or alignment parameters (Table 2).

**Table 2:** Association between patient characteristics, alignment parameters, and the difference in varus-valgus laxity.

	β	95% CI	*p*-value
**Age**	0.03	-0.0004 to 0.06	0.05
**Sex**	-0.48	-1.12 to 0.17	0.14
**mHKA**	0.03	-0.09 to 0.16	0.58
**LDFA**	-0.01	-0.18 to 0.16	0.89
**MPTA**	0.02	-0.15 to 0.19	0.82

Difference in varus-valgus laxity was calculated by subtracting the valgus angle from the varus angle.

mHKA: mechanical hip–knee–ankle angle; MPTA: medial proximal tibial angle; LDFA: lateral distal femoral angle; CI: confidence interval

## Discussion

The most important finding of this study was that varus laxity was significant but only 0.7° greater than valgus laxity in healthy Japanese population. Additionally, contrary to our hypothesis, laxity did not correlate with alignment parameters. Despite diverse lower limb alignments, the study found that ligament balance asymmetry remained relatively consistent, suggesting that ligament balance was reliable across different alignments, which may enhance clinical assessments and treatment strategies.

We have summarized previous reports on native knee laxity [8, 14–20] in Table 3. The difference in varus-valgus laxity varies between reports, ranging from 0.3–0.5° to >2°. Several factors may have contributed to this discrepancy.

**Table 3:** Summary of past reports regarding native knee laxity.

Author	*In vivo or in vitro*	Number of knees	Age (years)	Sex (male: female)	Method	Applied force	Knee Extension angle	Measurement (absolute or relative)	Varus	Valgus
**Okazaki et al. [8]**	*In vivo*	50	25.9 ±7.5	34:16	Stress Radiograph	15 kg	10°	Absolute	4.9° ±2.0°	2.4° ±1.6°
**Heesterbeek et al. [16]**	*In vivo*	30	62 ±6.4	15:15	Stress Radiograph	15 Nm	0°	Relative	2.8° ±1.3°	2.3° ±0.8°
**Deep et al. [14]**	*In vivo*	267	26.2	155:112	Navigation	10 Nm	0°	Relative	Men	
									3.0° ±1.8°	4.2° ±2.0°
									Women	
									3.3°±2.2°	5.0° ±2.4°
**Yoo et al. [20]**	*In vivo*	200	39 ±11.9	100:100	Custom made device	Manual stress	20°	Absolute	7.0° ±1.2°	4.1° ±0.9°
**Shultz et al. [17]**	*In vivo*	20		10:10	Custom made device	10 Nm	20°	Absolute	6.9°	5.2° ±1.0°
**Te Molder et al. [18]**	*In vivo*	40	62.7 ±5.8	13:27	stress Radiograph	15 Nm	0°	Relative	Men	
									1.5° ±0.9°	2.0° ±0.6°
									Women	
									2.4°±1.3°	2.1°±1.3°
**Gladnick et al. [15]**	*In vitro*	20	45 ±14	14:6	Robotic testing device	4 Nm	0°	Relative	2.0°±1.1°	1.5°±0.5°
**VanDamme et al. [19]**	*In vitro*	12	68	-	Navigation	9.8 Nm	0°	Absolute	3.1±0.8 mm	2.6±1.0 mm

First, the measurement of joint angle changes during stress radiographs can yield different results depending on whether the angles are calculated as absolute or relative to the neutral position [21]. Absolute measurements reflect the joint surface’s inclination angle, whereas relative measurements indicate ligament extensibility. Due to individual variations in joint surface inclination at the neutral position, substantial differences between absolute and relative values can be observed. Therefore, when evaluating “ligament balance,” relative values that reflect ligament extensibility may provide more representative information about the ligament’s condition, minimizing the influence of joint structure.

Second, the knee joint flexion angle during a stress radiograph can affect the results. The reason for slightly flexing the knee during a stress radiograph was to place the tibial plateau perpendicular to the film in the report of a healthy knee [8] or to minimize the influence of flexion contracture in the analysis of postoperative knee [21–24]. However, previous reports revealed that the values differ at 0° and 15° flexion [14, 15], and the greatest change in laxity occurred between 0–10° flexion [25]. Coupled motion during knee flexion also plays a role, as rotational coupled motion occurs with varus-valgus stress at flexion angles >15°, whereas it is minimal at full extension [15]. Therefore, even slight flexion affects the results of varus-valgus stress radiographs. Evaluating ligament balance at full extension remains relevant and important for healthy knees, as it minimizes these variations and ensures a stable and reproducible assessment of ligament balance.

Considering these influences, this study indicates that the difference in varus-valgus laxity is generally small, which is clinically close to a rectangle when measured at full extension using relative calculation. Variations observed in previous studies appear to be largely due to differences in measurement methods rather than inherent factors. Discrepancies between studies can be minimized by carefully aligning measurement conditions, such as ensuring consistent knee flexion angles and using relative calculations. This underscores the importance of methodological consistency in achieving more reliable and comparable results across studies.

The lower limb alignment of the Japanese population in this study averaged mHKA as-1.9, which was more varus, compared with the lower limb alignment in Western populations (mHKA = -1.0–1.3)as per previous reports [26–28]. Furthermore, the distribution of lower limb alignment in this study was diverse, with the maximum varus angle exceeding -9°. These observations have recently been increasingly recognized as part of the concept of constitutional varus [26]. However, this study did not demonstrate a correlation between ligament balance and lower limb alignment despite this diversity.

A previous study reported that female individuals generally exhibit greater laxity than male individuals [29]. However, when evaluating varus-valgus difference as “ligament balance,” no significant sex differences or associations with alignment were observed, as indicated in this study. Thus, ligament balance shows consistency regardless of sex or alignment variations. To our knowledge, this study is the first to investigate the correlation between ligament balance and alignment. In recent TKA, which aims to reconstruct the pre-arthritic joint and ligament balance, understanding the relationship between alignment and ligament balance, as demonstrated in this study, is important.

This study has some limitations. First, although the sample size in this study was larger than that in previous reports and we conducted a sample size calculation before the study, it may still lack sufficient representation of the diverse spectrum of skeletal morphologies. Second, previous studies have demonstrated a correlation between HKA and extension gap asymmetry in OA knees [30–34], and another study revealed that ligament balance might change with OA progression [35]. In contrast, our study focused on healthy knees and suggested that ligament balance asymmetry was not inherent in healthy knees but may develop with OA progression. Consequently, the direct applicability of our results to TKA cases remains uncertain. Future research should explore how ligament balance changes during OA progression to evaluate the applicability of our findings to TKA cases. Finally, we only evaluated ligament balance in extension due to the difficulty in stress imaging and the substantial impact of coupled motion during flexion. Stability in mid to full flexion is critical in TKA; however, assessing flexion stability using a highly reproducible i*n vivo* method that minimizes the influence of coupled motion is important.

In conclusion, this study demonstrates that healthy knees of Japanese individuals exhibit significantly greater varus laxity than valgus laxity (0.7°). No correlation was observed between ligament balance and lower limb alignment. These findings underscore the importance of recognizing alignment diversity while noting that ligament balance is not influenced by alignment. This finding is particularly relevant in modern TKA techniques that focus on patient-specific joint and ligament reconstructions.

## Supporting information

S1 Data (XLSX)
